# Prevalence of Crimean-Congo Hemorrhagic Fever Among High Risk Human Groups

**DOI:** 10.5812/ijhrba.11520

**Published:** 2014-03-05

**Authors:** Batool Sharifi-Mood, Maliheh Metanat, Roya Alavi-Naini

**Affiliations:** 1 Infectious Diseases & Tropical Medicine Research Center, Zahedan University of Medical Sciences, Zahedan, IR Iran

**Keywords:** Hemorrhagic Fever, Crimean, Prevalence, Dangerous Behavior

## Abstract

**Background:**

Crimean-Congo hemorrhagic fever (CCHF), an acute viral infection, is a zoonotic disease which is transmitted to humans by infected ticks, direct contact with fresh meat or blood of infected animals (usually domestic livestock), or direct contact with the blood or secretions of an infected person. Livestock handlers, skin processors, veterinary staff, livestock market workers, and other personnel engaged in jobs requiring some contact with animals and/or animal products are at high risk for CCHF. Most reported cases of this disease in Iran belong to butchers and slaughterhouse workers.

**Objectives:**

We aimed to study the prevalence of CCHF in slaughterhouse workers and livestock handlers who were admitted to Boo-ali Hospital for treatment of CCHF.

**Materials and Methods:**

We evaluated all patients’ files with confirmed CCHF admitted to Boo-ali Hospital in Zahedan, in southeastern part of Iran, during 1999-2011. Then, we examined the prevalence of disease among the high risk groups.

**Results:**

Out of 362 patients with CCHF (86% male, 14% female; with age range 12-78 years), 123 (34%) were slaughterhouse workers, 103 (28.5%) livestock handlers and farmers, 32 (9%) housewives, 7 (2%) students, 6 (1.9%) teachers, 4 (1.2%) military personnel, and other groups were workers with different employments.

**Conclusions:**

The present study showed that CCHF is highly prevalent in high risk occupational groups in Zahedan, Iran. Further surveillance, teaching and prevention programs are recommended.

## 1. Background

Crimean-Congo hemorrhagic fever (CCHF), is an acute viral infection, caused by CCHF virus (genus *Nairovirus*, in the family Bunyaviridae). The disease has received great consideration because of its relatively high mortality rate (20-70%), especially when there is a known risk factor. It is an endemic disease in Iran, especially in the southeastern part of the country, and large outbreaks have occurred during the spring and summer seasons since 1999.

CCHF is a zoonosis disease transmitted to large and small mammals and even birds by ticks (1-3). The main group of vectors involved in CCHF virus transmission appears to be ticks of the genus *Hyalomma*. Immature ticks get the virus by feeding on small infected vertebrates. They remain infected throughout their metamorphosis and after maturity, transmit the infection to domestic animal stock. Infected sheep, goats and cattle develop high titers of virus in their blood, but are usually asymptomatic. Transovarian transmission has also been demonstrated. Birds are generally immune with the exception of ostriches (1, 2, 4, 5). 

The virus is transmitted to humans by infected ticks, direct contact with fresh meat or blood of viremic animals (usually domestic livestock), or direct contact with the blood or secretions of an infected human (1, 2). Animals become viremic one week after infection with only a mild to moderate fever, which is often unnoticed. Humans appear to be the only vertebrate species in which CCHF virus precipitates serious and often fatal disease (1, 6-8). Sheep and goats are the largest domestic animals, which live next to humans. Researchers showed that even sheep that were infected previously and have already had anti-CCHF virus IgG could be re-infected and transmit the virus (8-10).

Humans could be infected through the skin contact, ingestion of the under-cooked/raw meat, or drinking unpasteurized milk. Aerosol transmission is also reported in a few cases in Russia. Human-to-human transmission can also occur, particularly when skin or mucous membranes are exposed to infected blood or secretions (11). Butchers were more likely to have CCHF antibody than people with other job groups. Increasing human exposure to infected animals raises the risk of infection. Livestock handlers, skin processors, veterinary personnel, livestock market employees, and other personnel engaged in jobs requiring some contact with animals and/or animal products are at high risk (9-11). Many studies have demonstrated that most human infection in Iran has taken place among people having close contact with the blood, secretions, and tissues of infected livestock (1, 2, 4, 8-10). Also, a report from pakistan (in 2002) revealed that most infections were observed among people employed in the industry of keeping and slaughtering of livestock (11).

## 2. Objectives

We aimed to study the prevalence of CCHF among those admitted to Boo-ali Hospital for the treatment of CCHF.

## 3. Materials and Methods

In this cross-sectional and retrospective study, we evaluated all patients’ files with confirmed CCHF admitted to Boo-ali Hospital (Zahedan, southeast of Iran) during1999-2011. The questionnaire included questions on demographic characteristics, uncooked or raw meat consumption, preparation practices, and risk factors. After collecting data, the prevalence of disease among patients was evaluated according to their jobs.

## 4. Results

Out of 362 patients with CCHF (86% male, 14% female; with age range 12-78 years) 123 (34%) were slaughterhouse workers, 103 (28.5%) were livestock handlers and farmers, 32 (9%) were housekeeper women, 7 (2%) were students, 6 (1.9%) were teachers, 4 (1.2%) were military personnel and 87 (24%) were from other occupational groups including building workers, grocers, and cooks (Table 1). 

**Table 1.** Frequency of CCHF According to Job^a^

Job	Slaughter-house Worker	Livestock Handler-farmer	Housewife	Student	Teacher	Military Staff	Other
**Male**	112 (31)	99 (27.3)	0 (0)	7 (2)	3 (0.8)	4 (1.1)	87 (24)
**Female**	11 (3)	4 (1.1)	32 (8.8)	0 (0)	3 (0.8)	0 (0)	0 (0)
**Total**	123 (34)	103 (28.4)	32 (8.8)	7 (2)	6 (1.6)	4 (1.1)	87 (24)

^a^ No. (%).

## 5. Discussion

This cross-sectional study was conducted to investigate the prevalence of CCHF in slaughterhouse workers and livestock handlers admitted to Boo-ali Hospital in Zahedan, southeast of Iran. In our study, the prevalence of CCHF infection among slaughterhouse workers, butchers, and handler livestock-farmers was 62.5%. This rate of infection is high compared to the 5% infection rate among the slaughterhouse workers in Isfahan province, which has the second highest rate of reported cases of this disease in Iran (8). 

Most reported cases are the result of occupational exposure. CCHF is common in shepherds, farmers, veterinarians, abattoir workers, and laboratory workers. Health-care personnel (HCP) are also at high risk, particularly after exposure to the patient’s blood and secretion. According to a nosocomial outbreak in South Africa, 33% of HCP exposed via needle stick injuries became ill. Nine percent of those who had other forms of contact with infected blood also developed CCHF (11).

The highest rate of this infection has been reported in Sistan and Baluchestan, a province in the Southeast of Iran - in the border of Afghanistan and Pakistan - where the rate of infected imported livestock to Iran is high. Clinical disease is rare in infected mammals, but is usually severe in infected humans. Outbreaks of illness are usually attributable to handling infected animals or people.

Many studies have showed that slaughterhouse workers, butchers, and livestock handlers are high-risk groups for CCHF (2, 8-12). Only, one study conducted in Yasuj (Iran) reported that the seroepidemiology of Crimean-Congo hemorrhagic fever is not prevalent in high risk professions (13). In the Yasuj survey, out of 108 subjects at risk (34 butchers, 20 slaughterers, 14 slaughterhouse workers, 20 waiters, and 20 housewives), 5 cases (4.6 percent) were found with positive serology among restaurant workers and slaughterers. There was no positive serology in the other groups.

In a study from South Africa, 15 cases of CCHF were reported and all of them were workers in ostrich slaughterhouses (14). In a report from Oman, in the Middle East, of the individuals working in animal contact-related jobs, 73 (30.3%) of 241 non-Omani citizens and only 1 (2.4%) of 41 Omani citizens were CCHF antibody-positive (15). Butchers were more likely to have CCHF antibody than people in other job categories. None of the 74 antibody-positive individuals in this study answered yes to having ‘ever been hospitalized for CCHF or an illness with fever and bleeding’; a finding which suggests a greater proportion of subclinical than clinical disease in humans. It is estimated that the ratio of asymptomatic to symptomatic patients is 5:1 (15). A report from Mauritania demonstratedthat half of the patients with this disease were slaughterhouse workers and butchers, which suggests that the primary mode of animal-to-human transmission was direct contact with blood of infected animals (9).

The higher incidence of high risk behavior and the failure to observe personal protection according to surveillance system and prevention program in novice butchers and slaughterhouse workers are important factors responsible for their increased rate of infection compare to older people (15, 16). 

In the present study, like previous studies, the highest rate of infection was found in younger men (age range of 21 to 40) (12, 17). This age range is associated with more activities and hence, the probability of more contact with infected livestock in jobs such as slaughtering, skin processing, and livestock handling.

We should also remember the long borders of Sistan and Baluchestan province with Afghanistan and Pakistan and the likelihood of more imported infected livestock from these countries which could be a way for more spread of diseases from this region to other parts of our country. Therefore, the borders need to be controlled and quarantine practices to be enforced to prevent human exposure and ongoing dissemination of infected ticks and livestock in this region. Enforcing acaricidal treatment and quarantine on the importer are the important methods for control of infection before entering into the country when ticks are present on animals. Most importantly, taking precautions such as gloving and booting to reduce skin contact and percutaneous exposure of humans is essential.

In endemic areas, prevention measures include avoiding ticks and contact with infected blood or tissues. Clothing must prevent tick attachment; long pants tucked into boots and long-sleeved shirts are recommended. Acaroids should be used on livestock and other infected animals to control ticks, particularly before slaughter or export. Unpasteurized milk should be avoided. CCHFV in meat is usually inactivated by cooking at 60°C for 30 minutes. Laboratory staff must follow biosafety precautions and viral isolation techniques should be carried out in laboratories where biosafety level 4 is available. CCHFV can be inactivated by disinfectants, including 1% hypochlorite and 2% glutaraldehyde. 

Prophylactic treatment with ribavirin has occasionally been used after high-risk exposures. The direct contact cases should be monitored for at least 14 days since the last contact with the patient or other sources of infection and their temperature should be checked twice daily. If the case develops a temperature ≥ 38.5°C, headache, and muscle pain, he/she must be admitted to a hospital, and treated with ribavirin. Another way for prevention of many infectious diseases is vaccination. Since the 1970s, several vaccine trials around the world against CCHF have been scrapped due to high toxicity or ineffectiveness. In 2011, a Turkish research team has successfully developed the first non-toxic preventive vaccine, which passed clinical trials. This vaccine is pending approval by the FDA (18).

Our study showed that CCHF is highly prevalent in high risk occupational groups in Zahedan, Iran. Therefore, further surveillance systems and prevention programs are recommended. Where mammalian tick infection is common, agricultural regulations are required for de-ticking farm animals before transportation or delivery for slaughter. Personal tick avoidance measures are also recommended, such as the use of insect repellents, adequate clothing and body inspection for adherent ticks. When feverish patients with evidence of bleeding require resuscitation or intensive care, body substance isolation precautions should be taken. Finally, special stocks of ribavirin must be stored to protect high risk personnel against CCHF.
